# Effects of coumaphos and imidacloprid on honey bee (Hymenoptera: Apidae) lifespan and antioxidant gene regulations in laboratory experiments

**DOI:** 10.1038/s41598-018-33348-4

**Published:** 2018-10-09

**Authors:** Ales Gregorc, Mohamed Alburaki, Nicholas Rinderer, Blair Sampson, Patricia R. Knight, Shahid Karim, John Adamczyk

**Affiliations:** 1 Mississippi State University, Center for Costal Horticulture Research, Poplarville, MS USA; 2 Agricultural Institute of Slovenia, Ljubljana, Slovenia and University of Maribor, Faculty of Agriculture and Life Sciences, Maribor, Slovenia; 3 The University of Southern Mississippi, Department of Biological Sciences, Hattiesburg, MS USA; 4 USDA, ARS, Thad Cochran Southern Horticultural Research Laboratory, Poplarville, MS USA

## Abstract

The main objective of this study was to test comparatively the effects of two common insecticides on honey bee *Apis mellifera* worker’s lifespan, food consumption, mortality, and expression of antioxidant genes. Newly emerged worker bees were exposed to organophosphate insecticide coumaphos, a neonicotinoid imidacloprid, and their mixtures. Toxicity tests were conducted along with bee midgut immunohistological TUNEL analyses. RT-qPCR assessed the regulation of 10 bee antioxidant genes linked to pesticide toxicity. We tested coumaphos at 92,600 ppb concentration, in combination with 5 and 20 ppb imidacloprid. Coumaphos induced significantly higher bee mortality, which was associated with down regulation of catalase compared to coumaphos and imidacloprid (5/20 ppb) mixtures, whereas, both imidacloprid concentrations independently had no effect on bee mortality. Mixture of coumaphos and imidacloprid reduced daily bee consumption of a control food patty to 10 mg from a coumaphos intake of 14.3 mg and 18.4 and 13.7 mg for imidacloprid (5 and 20) ppb, respectively. While coumaphos and imidacloprid mixtures induced down-regulation of antioxidant genes with noticeable midgut tissue damage, imidacloprid induced intensive gene up-regulations with less midgut apoptosis.

## Introduction

Many pesticides used in agriculture are harmful, not only to target pests, but also to beneficial arthropods, including pollinating bees^1^. Some pesticides such as acaricides are purposely placed into honey bee colonies for Varroa control. When these contaminated bee foragers return to the colony, they inadvertently expose hive bees to toxic pesticide residues. Exposure to persistent pesticides through social interaction is one of the many suspected causes of colony collapse disorder (CCD), along with other co-mortality and co-morbidity factors such as pathogens, parasites, stress, and starvation^2,3^. All of these factors, combined with low genetic diversity within honey bee colonies, may operate separately or synergistically to reduce population size^4^. Further study is needed to ascertain which specific factors or combination of factors affect worker bee survival^5^. Controlled cage experiments that simultaneously manipulate a wide range of independent variables known to influence the survival of non-captive bees can simulate any sub-lethal effects of common insecticides such as coumaphos and imidacloprid on worker behavior and survival.

Coumaphos, beside the neonicotinoids, is one of the main pesticides of concern for honey bee health. Coumaphos is a widely available organophosphate-based acaricide formulated by Bayer into CheckMite strips and Perizin®. Beekeepers introduce these coumaphos products into colonies to control parasitic varroa mites (*Varroa destructor*). Coumaphos acts systemically with small amounts of coumaphos being eaten by bees and spread trophalactically. Yet, the bulk of the acaricide is distributed colony-wide within 3 h through dermal contact among nestmates^6,7^. Consequently, acaricides like coumaphos are external, nonpathogenic factors widely used in beekeeping and, as such, coumaphos residues are often found in sundry hive products: bee bread, propolis, wax, comb and royal jelly, the latter being a vital foodstuff for brood and queen rearing^8^. In a recent survey of three migratory beekeeping operations experiencing suspected CCD, metabolites of 121 pesticides applied on flowering apple were found, with coumaphos (1.0 μg/kg to 91.9 mg/kg) found in 98% of wax samples^9^. Other studies confirm the presence of coumaphos residues in hive products such as wax, honey and propolis^10–12^. The acute lethal dose (LD_50_) of coumaphos for individual bees varies from (3–6) μg, with lower doses proving more toxic to older bees^6^. Chronic exposure to coumaphos can result in reduced foraging activity^13^, affect the size of hypopharyngeal glands, and increase the level of programmed cell death within bee tissues^8^. Although coumaphos is regarded as being weakly toxic to honey bees^14^, more study is needed to determine whether this acaricide decreases individual bee survival and hence the health of the pollinator workforce.

Pesticide residues acquired by bee foragers outside of the colony are also of concern. We now know that a variety of pesticides used in honey bee colonies can have adverse, sublethal effects on individual bees and their tissues^15^. Honey bees may also contact insecticides applied to crops both before and during crop bloom. Outside of colonies, honey bees are becoming increasingly exposed to imidacloprid, a systemic, neurotoxic insecticide commonly applied aerially or as soil drenches and seed treatments in agronomic and horticultural crop fields^16,17^. Neonicotinoids at sub-lethal doses were shown to impair the olfactory memory of honey bees and their learning capacity^18,19^. Moreover, this particular class of pesticides was actively linked to gene regulation of many honey bee detoxification genes that metabolize toxic molecules or minimize their fatal effects on the organism^20–22^. Imidacloprid, and related neonicotinoids, thiamethoxam and clothianidin, are acutely toxic to honey bees with acute oral LD_50_ ranging from (0.004–0.005) μg per bee^23^, rates often greatly exceeding those in the pollen and nectar of seed-treated corn, sunflowers, and rape (i.e., 2–3.9 μg/kg in pollen and less than 2 μg/kg in nectar)^24–26^. Characteristics of imidacloprid toxicity in honey bees have been investigated using both dermal and oral bioassays^27,28^. The mode of action of imidacloprid involves its agonistic binding to post-synaptic nicotinic acetylcholine receptors in the insect’s central nervous system^29^. Thus, its neurological symptoms appear as hyperactivity, tremors, paralysis and finally death. Imidacloprid also induces cell death in adult bees and is associated with reductions in the diameter of the acinae of the hypopharyngeal glands, glands necessary for producing royal jelly^8^.

Therefore, the objectives of this study were (1) to evaluate bee mortality rate chronically exposed to varying concentrations of coumaphos through their diet, (2) to study how chronic exposure to such compounds induces physiological stress on bees and regulates antioxidant genes, and (3) to monitor changes in midgut morphology and cell death due to increasing coumaphos concentrations and any possible interaction with simultaneous imidacloprid exposure at realistic field rates. To our knowledge, this report represents the first assessment of the effects of sub-lethal doses of coumaphos and imidacloprid on individual honey bee workers using combined toxicological, gene expression, and cell death immunohistological analyses.

## Results

### Coumaphos effects on bee survival (Experiment 1)

**Figure 1:**
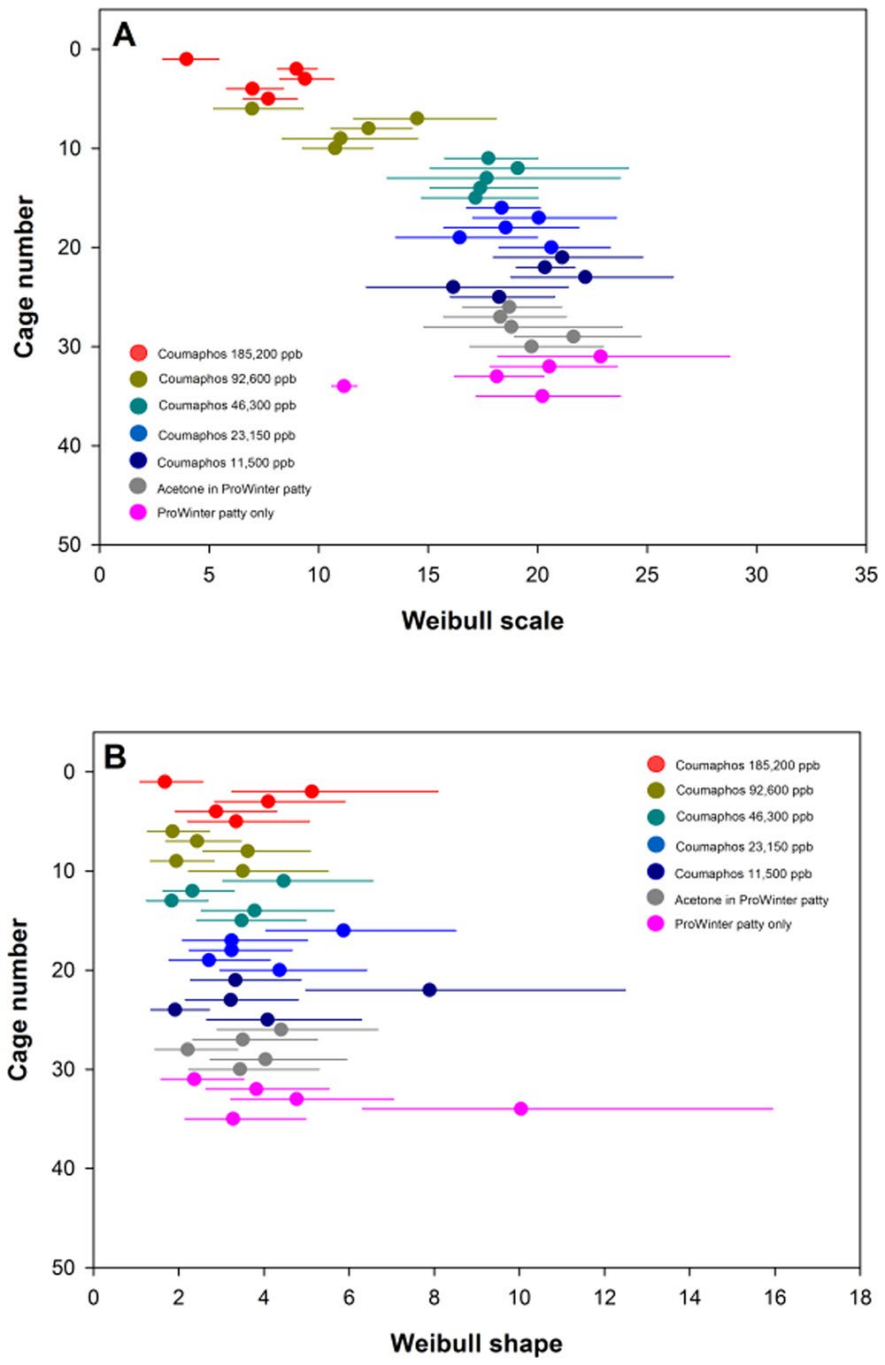
Survivorship analysis for bees fed various concentrations of coumaphos in laboratory bioassay. The Weibull scale parameter (d) (**A**) and shape (**B**) with censoring fit for all cages is shown. Treatments were coumaphos mixed in Pro Winter sugar patty at 185,200 ppb; 92,600 ppb; 46,300 ppb; 23,150 ppb and 11,500 ppb. Controls were acetone treated patty and patty alone. Solid dot indicate parameter estimate and lines show confidence limits (α = 0.05). Confidence limits that overlap are not considered significantly different from one another.

Caged bees exposed to coumaphos concentrations of 185,200; 92,600; 46,300; 23,150 and 11,500 ppb remained active throughout the experiment. Routine worker behavior such as grooming appeared normal for bees across the seven treatment groups, including the behaviors of bees exposed to the highest coumaphos concentrations. At the observation times, when dead bees were sampled, no differences were observed in comparison to control-untreated bees of bees exposed to acetone as diluent added to patty. There were significant differences among the treatments (P < 0.05). Bees fed concentrations of coumaphos at 185,200 and 92,600 ppb had significantly higher mortality than did each of the lower coumaphos concentrations or either controls (Table 1). The three lower coumaphos concentrations (46,300; 23,150 and 11,500) ppb were relatively non-toxic to bees when compared with untreated control bees. The scale and shape for the means (d) to reach the 30, 50, and 70^th^ mortality percentile for each cage among a given treatment is shown in Fig. 1.

**Table 1:** Mortality of honey bees treated in laboratory bioassays with coumaphos at various doses.

Treatment group (Coumaphos (ppb)	Mean Percentile (d)		
	30^th^	50^th^	70^th^
185,200	5.46 b	6.63 c	7.82 c
92,600	7.43 b	9.61 b	11.93 b
46,300	12.47 a	15.66 a	19.01 a
23,150	14.20 a	17.00 a	19.78 a
11,500	14.60 a	17.62 a	20.70 a
Acetone + Control Patty	14.31 a	17.41 a	20.54 a
PrpWinter Control Patty	14.98 a	16.77 a	19.58 a
***ANOVA***			
df	6, 24	6, 24	6, 24
F-value	15.52	20.65	19.53
P-value	<0.001	<0.001	<0.001

The mean (d) for each treatment to reach the 30, 50, and 70^th^ mortality percentile is shown. Experiment was terminated at day 40. Survival analysis was conducted using the LifeReg procedure and treatment differences analyzed using ANOVA with the Glimmix procedure (SAS version 9.4). Means within a column followed by a common letter are not significantly different from one another (LSMEANS; α = 0.05).

### Coumaphos and imidacloprid interaction (Experiment 2)

**Figure 2:**
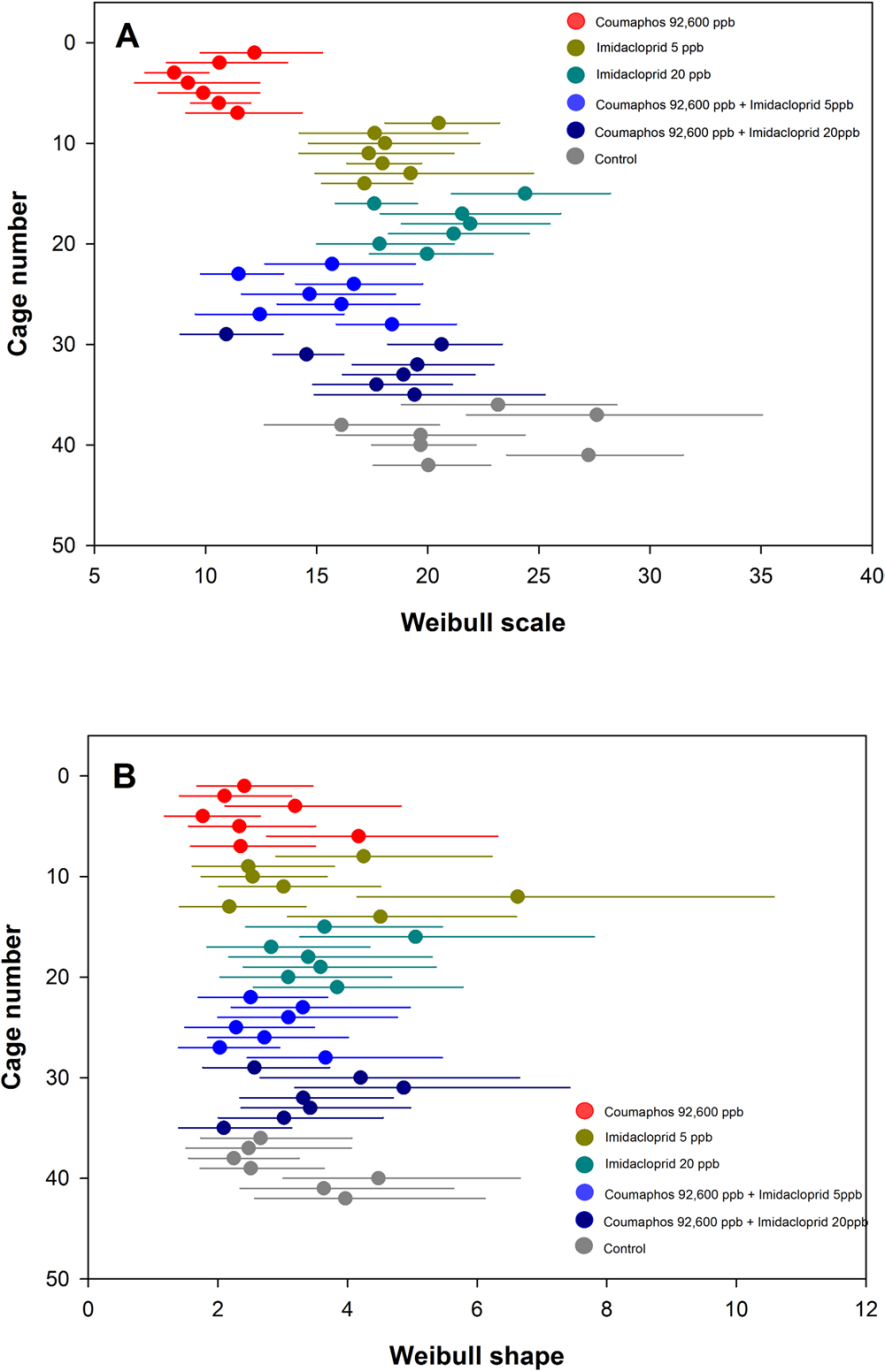
Survivorship analysis for bees fed coumaphos, imidacloprid, and combinations of both in laboratory bioassay. The Weibull scale parameter (d) (**A**) and shape (**B**) with censoring fit for all cages is shown. Treatments were coumaphos mixed in Pro Winter sugar patty at 92,600 ppb, imidacloprid at 5 and 20 ppb, and coumaphos mixed with both doses of imidacloprid. Control was patty alone. Solid dot indicate parameter estimate and lines show confidence limits (α = 0.05). Confidence limits that overlap are not considered significantly different from one another.

Combinations of coumaphos (92,600 ppb) and imidacloprid treatments offered to caged bees had significantly different effects on survivorship (P < 0.05) (Table 2). Bees offered coumaphos had significantly higher mortality at the 30, 50, and 70^th^ percentile compared to both concentrations of imidacloprid as well as both mixtures of coumaphos and imidacloprid. In fact, coumaphos was highly toxic to bees killing nearly than half of them by day 9. The two low doses of imidacloprid (5 ppb and 20 ppb) were proven to be relatively non-toxic to honey bee workers. However, mortality for bees fed imidacloprid and coumaphos combinations were often significantly lower than bees fed coumaphos alone. The scale and shape for the means (d) to reach the 30, 50, and 70^th^ mortality percentile for each cage among a given treatment is shown in Fig. 2.

**Table 2:** Mortality of honey bees treated in laboratory bioassays with coumaphos (96,000 ppb), imidacloprid at 5 and 20 ppb, and combinations of coumaphos (96,000 ppb) and imidacloprid.

Treatment group	Mean Percentile (d)		
	30^th^	50^th^	70^th^
Coumaphos	6.83 d	8.93 e	11.18 e
Imidacloprid 5ppb	13.29 ab	16.29 bc	19.36 bc
Imidacloprid 20ppb	15.39 a	18.58 ab	21.75 ab
Coumaphos + Imidacloprid 5ppb	10.35 c	13.18 d	16.13 d
Coumaphos + Imidacloprid 20ppb	12.56 bc	15.48 cd	18.43 cd
ProWinter Control Patty	15.53 a	19.38 a	23.35 a
***ANOVA***			
df	5, 36	5, 36	5, 36
F-value	14.81	16.63	15.62
P-value	<0.001	<0.001	<0.001

The mean (d) for each treatment to reach the 30, 50, and 70^th^ mortality percentile is shown. Experiment was terminated at day 33. Survival analysis was conducted using the LifeReg procedure and treatment differences analyzed using ANOVA with the Glimmix procedure (SAS version 9.4). Means within a column followed by a common letter are not significantly different from one another (LSMEANS; α = 0.05).

### Food consumption

**Figure 3:**
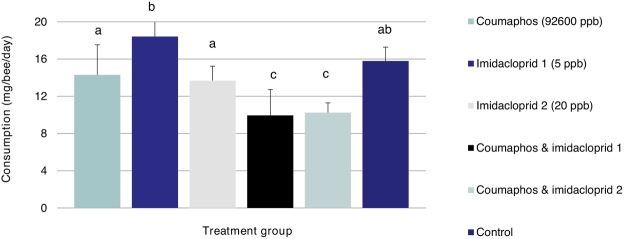
Consumption rate of Pro Winter sugar patty amended with or without coumaphos and imidacloprid insecticides, or their combinations. Letters that are the same indicate means that are not significantly different according to the Tukey HSD test (P < 0.05). Bars indicate mean +1 standard deviation.

The highest patty consumption rates per bee were observed in bees fed with imidacloprid 5 ppb, followed by the control-untreated bees (15.79 ± 1.49 mg/bee/day). On the other hand, the lowest consumption rates were recorded in both coumaphos/imidacloprid mixtures, for coumaphos + imidacloprid 5 ppb and for coumaphos + imidacloprid 20 ppb. Values are shown in Fig. 3. Adding imidacloprid at either concentration (5, 20) ppb to coumaphos significantly (P < 0.05) reduced food consumption by approximately 25% when compared to coumaphos independently and by 33% compared to the untreated control.

### Antioxidant gene expression

**Figure 4:**
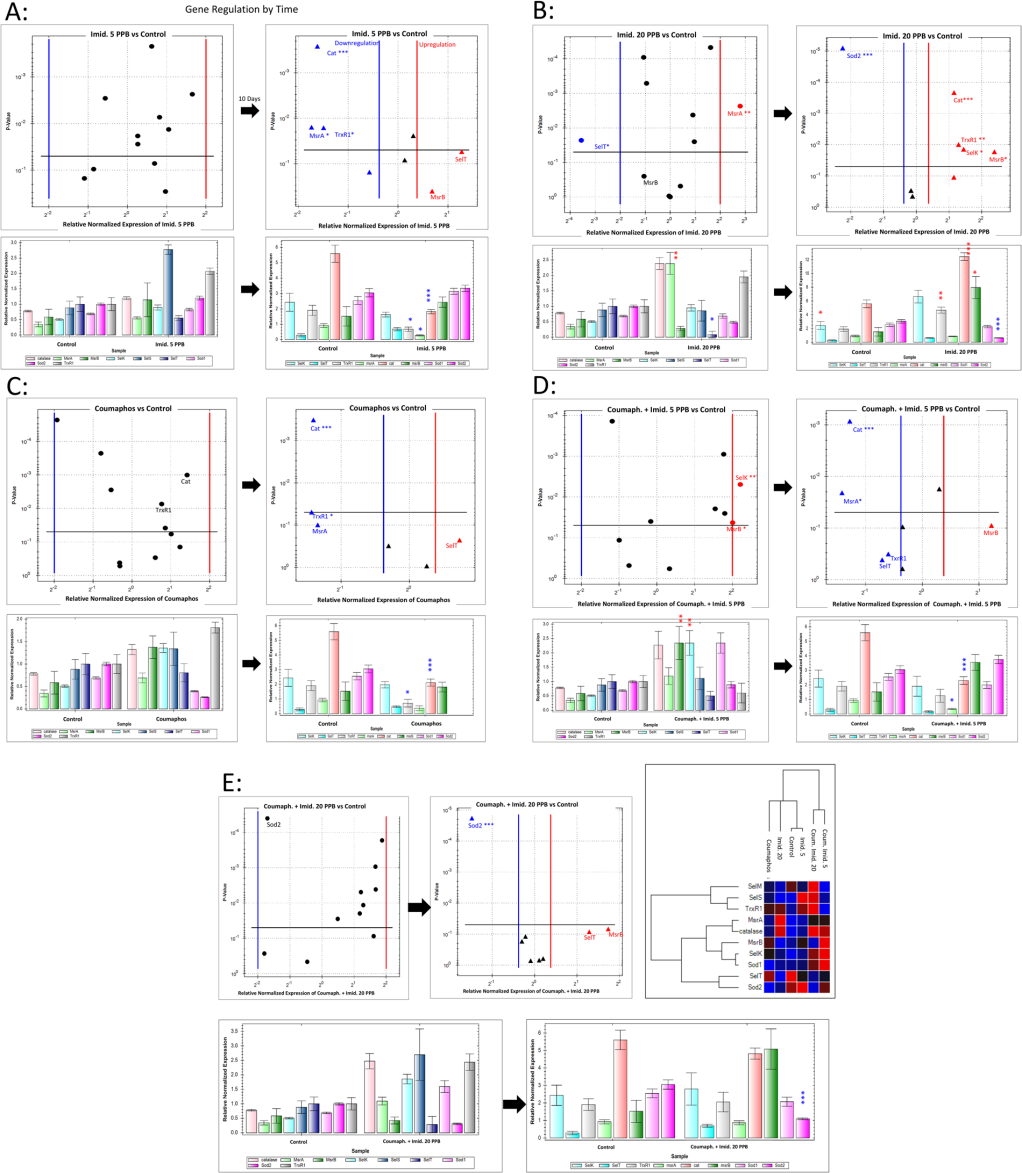
Volcano plots and bar charts of the relative expression of target genes in caged-bees through time and exposure to pesticides. (**A**) represents the difference in the studied gene expression versus the untreated control after 10 days of feed with Imidacloprid (5 ppb); (**B**) Imidacloprid (20 ppb); (**C**) Coumaphos (92,600 ppb); D: Coumaphos and Imidacloprid 5 PPB; E: Coumaphos and Imidacloprid 20 PPB. All gene studies were normalized using the housekeeping genes mentioned in Table 3. The qPCR regulation threshold value was set at P = 0.05, gene expression below this value was not considered up or down-regulated. Level of significances are P < 0.05 *P < 0.01 **P < 0.001 *** and error bars are the Standard Errors SE.

The expression of 10 antioxidant genes varied significantly among treatments during the ten-day feeding period. Bees fed with 5 ppb imidacloprid for ten days showed significant down-regulations in three different target genes: Cat (P < 0.001), MsrA and TrxR1 (P < 0.01), Fig. 4A. Both SelT and MsrB genes expressed up-regulations but were not considered significant based on the normalization of our dataset while conducting the gene study, because they exceeded the critical P-value of 0.05. The higher concentration of imidacloprid (20 ppb) led to a significant up-regulation in catalase activity (Cat: P < 0.001) along with three other genes (TrxR1, SelK, MsrB; P < 0.05), while Sod2 was down-regulated (P < 0.001), Fig. 4B. The latter figure shows down and up regulations of the SelT and MsrA genes, respectively. Coumaphos showed no changes in the first sampling date (day 10), however, at day 20, two genes were down regulated (Cat; P < 0.001) and (TrxR1; P < 0.05), Fig. 4C. Cat and MsrA were both down regulated in bees fed for 10 days on coumaphos and 5 ppb imidacloprid mixture, Fig. 4D. The final treatment (coumaphos and 20 ppb imidacloprid) showed significant down regulation in a single target gene (Sod2; P < 0.001), Fig. 4E. Figure 4E’s Clustergram classified the data and generated a hierarchal tree based on the degree of similarity of expression for both target genes and treatments. This dendrogram revealed close similarities between (untreated control vs imidacloprid 5 ppb), (coumaphos vs imidacloprid 20 ppb), while the mixture of coumaphos and imidacloprid, regardless the imidacloprid concentrations, seemed to have similar effects on the regulation of the target genes, Fig. 4E.

### Immunohistochemical analysis of the midgut

**Figure 5:**
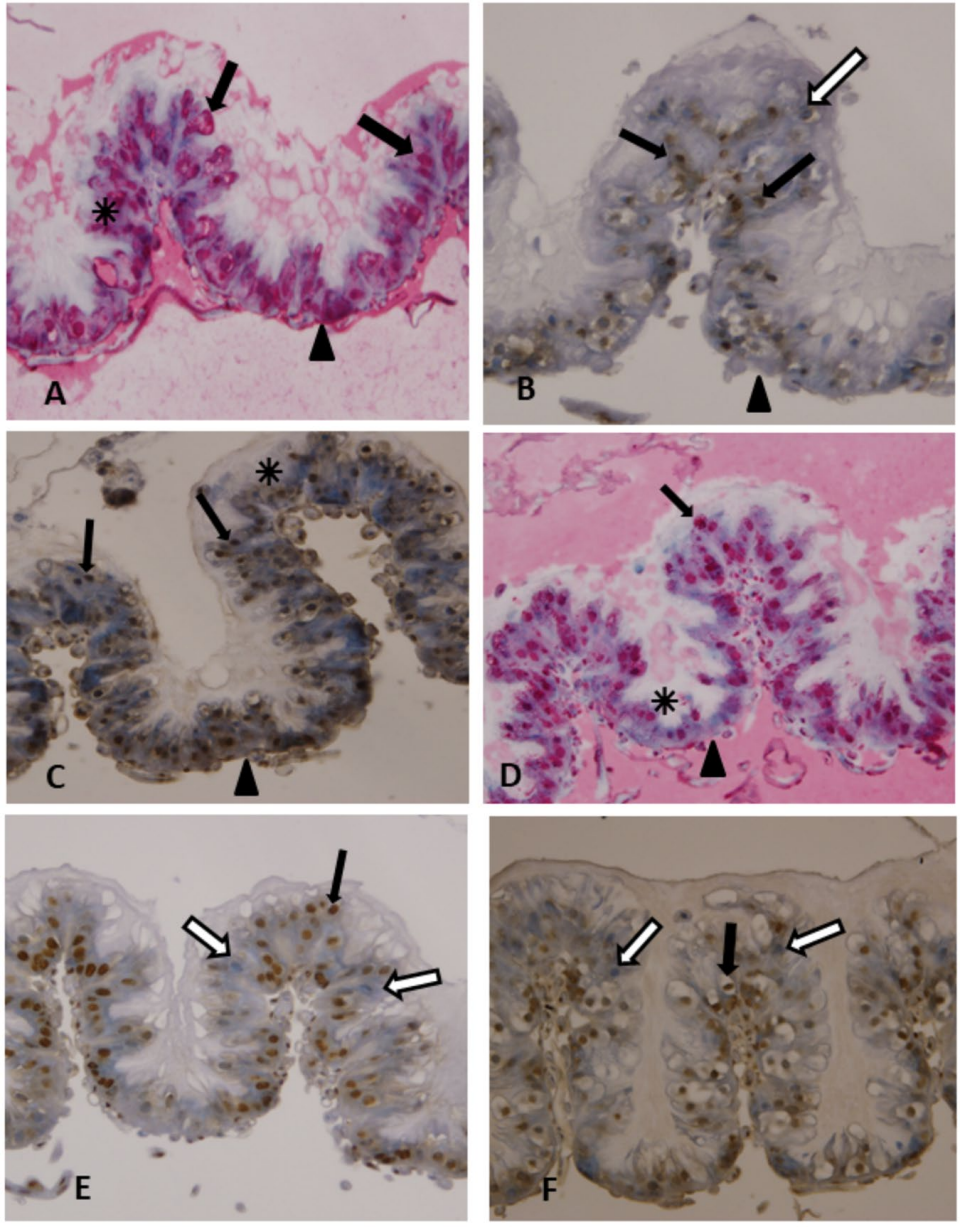
Ten-day-old bees exposed to 92,600 ppb coumaphos, 20 ppb imidacloprid or their combination. The figure shows dense red azo-dye staining localized in the nuclei (→) of midgut columnar cells. DAB (ApopTag) or fast red (ISCDDK) reaction product is localized to the cell nuclei (→), basal membrane of the epithelium (▼). Magnification: 400X, (**A**,**B**) bees exposed 10 days to coumaphos and imidacloprid; denote fast red reaction product is diffused throughout the vacuolated cell cytoplasm (). Denote enlarged perinuclear spaces. (**C**,**D**) Coumaphos treated bee, denote reduced thickness of the epithelium (); (**E**) imidacloprid treated bee; (**F**) untreated, control bee. Denote positive nuclei with brown reaction product (black arrow), and negative cells with blue nuclei (white arrow).

Control untreated bees exhibited the lowest level of cell death according to both ISCDDK and Apoptag indicators. However, in bees fed for 10 days on a diet containing both coumaphos and imidacloprid, the death of midgut epithelial cells exceeded that of the untreated control group. Notably, the proportion of TUNEL-positive nuclei in the midgut epithelial cells of bees fed coumaphos or imidacloprid was higher than the proportion of dead cells in bees with a combined coumaphos + imidacloprid diet. In bees fed coumaphos and imidacloprid mixture, their midgut epithelium hypertrophied whereas the cytoplasm of individual cells appeared less granular. In some apical portions of the midgut villi, cells lysed or contained enlarged perinuclear spaces, Fig. 5A. The red reaction product was also reduced in cells that had lysed or lost cytoplasm, Fig. 5B. TUNEL-positive cells detected with the ApopTag kit displayed less vacuolization, typical of cell secretion, Fig. 5C. In other portions of the midgut, cells appeared healthy (less to no reaction product) and no hypertrophy of the epithelium occurred, Fig. 5D. The shedding of cells during this period probably prevented further epithelial cell loss. In fact, midguts of bees exposed to coumaphos or imidacloprid and sampled at the end of the experiment had far fewer dead or dying cells than did midguts from bees fed an insecticide mixture or a control-untreated diet without acaricides as indicated by far fewer TUNEL-positive cells nuclei stained red using ISCDDK or brown using ApopTag. Individual coumaphos and imidacloprid treatments had the same effect (60–90% TUNEL-positive cells), compared to 20–55% TUNEL-positive cells for the coumaphos/imidacloprid combination, and only 5–15% TUNEL-positive cells for the control. Furthermore, better preserved epithelial structure were associated with apical extrusions typical for cell secretion and cell nuclei with brown reaction product, Fig. 5E. In the control untreated bees, the midgut epithelium layer was intact with apical cell extrusions into the midgut lumen and cytoplasmic vacuolization typical for cell secretions, Fig. 5F.

## Discussion

Coumaphos, an acaricide, and imidacloprid, a widely used crop insecticide, together can contaminate honey bee colonies through trophallaxis between nestmates and numerous other social interactions, which could eventually bio-accumulate and harm adult bees and brood^9^. In colonies treated with Perizin®, brood combs can contain anywhere between 1.8–43.4 mg/kg which is equivalent to 1,800–43,400 ppb of coumaphos^30^. The presence of coumaphos in wax consequentially reflects residues as high as 1,000–30,000 ppb in larvae and adult bees^11^. Coumaphos exhibited no anti-feedant properties in honey bees. In fact, bees are expected to eat small portions of coumaphos and orally spread the chemical colony-wide. In our bioassays, bees consumed coumaphos-treated patty at a rate of 14.3 mg patty/bee/day, a rate similar to if they were feeding on a coumaphos-free diet. Without any negative stimulus associated with coumaphos uptake, honey bees could easily receive lethal doses in a few days. For instance, in our bee bioassays, coumaphos did not impair bee feeding resulting in worker bees eating about 1.32 µg (a.i.) of coumaphos daily and receiving a minimal lethal dose within 3 days, i.e., LD_50_ of coumaphos ranges from 3 μg to 6 μg per bee^6^. Clearly, at rates below the LD_50_, coumaphos is unlikely to acutely kill bees, but a long-term exposure to coumaphos could result to chronic toxicity lowering bee longevity and hence lifetime productivity. We calculate honey bee colonies receive 2.78 g of coumaphos, impregnated into CheckMite strips for varroa mite control, a quantity potentially lethal to bees. Thus, coumaphos strips inside colonies could have harmful side effects on a subset of individual worker bees inside colonies, but the use of strips does far more damage to their intended targets, varroa mites. Our toxicity test results correspond to expression levels of the antioxidant genes linked to coumaphos-treated patty exposure. No gene regulations were recorded for any of the studied genes prior to day 10. At day 20 of the feeding process, only two antioxidant genes were down-regulated, Cat and TrxR1. Catalase actively protect cells from oxidative damage cause by reactive oxygen species (ROS) as illustrated in Figure S1, while TrxR1 produces two very strong thiol-based antioxidants, Trx and GSH. Their down-regulation in the case of coumaphos exposure may indicate little to no physiological stress imposed on bees by this particular acaricide.

In addition to its toxicity, sublethal side effects of coumaphos can include reduced trophallaxis, inadequate food storage, and finally, less active ingredient getting to varroa mites and hence weaker acaricidal activity^31^. More concerning is that the combined effect of coumaphos and trace amounts of an unrelated, but common crop insecticide, the neonicotinoid imidacloprid reduces food uptake by approximately 33%. Presence of 20 ppb imidacloprid in patty also reduces the uptake of coumaphos-treated patty in the same diet patty, and as a result, these bees had a lower rate of mortality along with an absence of any up regulation in antioxidant “stress” genes. Lower consumption in workers may still have an overall deleterious effect on colony growth, productivity, and overwintering survival. Imidacloprid, a common crop insecticide found in many hive products, by itself, is highly toxic to honey bees if applied incorrectly, and has an acute oral LD_50_ ranging from 4 × 10^−3^ to 5 × 10^−3^ μg per bee^23^. Moreover, imidacloprid above 20 μg/kg (20 ppb) can cause sublethal physiological and behavioral abnormalities in workers and queens^32–34^. Earlier studies, confirm that bulk feeding sublethal concentrations of 100 ppb imidacloprid to honey bees has no overt negative effects on colony development. Our bioassays identified sublethal effects of imidacloprid at concentrations as low as 5 ppb and 20 ppb on worker bees. Interestingly, honey bees exposed to 5 ppb imidacloprid within the hive exhibited greater foraging activity, while higher doses (20 and 100 ppb) did not^34^. Sublethal effects of imidacloprid that we observed profoundly affect bee health, both positively, by tempering the harmful effects of coumaphos, and negatively, by impairing food consumption by individual worker bees. Each caged bee feeding on 5 ppb imidacloprid consumes about 1 × 10^−4^ µg active ingredient and reaches a lethal dose within 13 days. At 20 ppb imidacloprid, a single bee consumes about 3 × 10^−4^ µg active ingredient per day and takes longer (40 days) to obtain a lethal dose because of this insecticide’s ability at a low concentration to extend bee longevity. For instance, for bees fed 5 ppb imidacloprid for 20 days, none of the antioxidant genes were up-regulated, while bees fed 20 ppb for the same duration induced significant physiological changes with up regulation of 5 genes including the catalase linked to oxidation as well as observed changes on the cellular level. Coumaphos interacting with imidacloprid greatly accelerates death in hive bees. For example, in colonies treated with coumaphos and imidacloprid, each bee hive receives ~ 0.25 µg coumaphos, 2 × 10^−4^ µg to 9 × 10^−4^ µg of the more toxic imidacloprid (a.i), and reaches its lethal dose within five days of feeding^23^.

Our results reveal the possible unforeseen consequences of interactions between a hive treatment for varroa (coumaphos) and a highly persistent crop insecticide (imidacloprid). The environmental residues of crop pesticides acquired by field bees will vary considerably. Imidacloprid and other neonicotinoid insecticides applied at recommended label rates to flowering crops are later found in field-collected pollen at concentrations ranging from 24–101 μg/kg and in nectar at concentrations from 7–16 μg/kg^35,36^. Rortais *et al*.^37^ estimate nectar-foraging honey bees are active 4 and 11 hour per day and consume 32–128 mg of carbohydrate per day. Concentrations of imidacloprid in pollen or nectar from seed-treated crops are reported to be below 5 μg/kg and are not lethal to honey bees under field conditions, but can cause sublethal effects in laboratory studies^23^. When bees are exposed to imidacloprid at levels higher than 20 μg/kg of food, ensuing physiological and behavioral abnormalities can include reductions in associative learning, queen fecundity, foraging activity, and increased susceptibility to other stressors^32,38^. At 100 ppb and 500 ppb of active ingredient in 50% sucrose solution, imidacloprid can reduce bee mobility and communicative capacity, with negative effects appearing after 30–60 minutes and subsiding after several hours^39^.

The different modes of action in interacting insecticides can cause not just neurological dysfunction, but also measurable cellular damage including sizeable lesions within the midgut wall. After ten or more days of feeding on coumaphos-treated patty and imidacloprid-treated patty, the epithelium lining of the bees’ midgut shows significant increases in the rate of apoptotic cell death, as well as increases in antioxidant activity in the case of imidacloprid-treated patty. Such damage may in fact be a defense mechanism whereby accelerated levels of apoptosis could prevent the spread of cellular damage to undamaged epithelium, a response akin to accelerated apoptosis in human T cells exposed to different classes of pesticides with alternative modes of action^40^. Lesions and losses in midgut tissue could lead to disease, starvation, and ultimately the death of worker bees. In coumaphos + imidacloprid-in patty treated bees, we observed a reduction in apoptotic cell death rate in the midgut with absence of any antioxidant up regulation and significant down regulations of three genes (Cat, MsrA and Sod2), but an increase in the cellular damage to midgut epithelium. Tissue degeneration associated with reduced gut epithelium evident in coumaphos and combined treated bees may explain bee death. Apoptosis observable with the TUNEL-technique is a form of programmed cell death in multicellular organisms, which involves a series of morphological changes including deformed cell membranes, cell shrinkage, chromatin condensation, and fragmentation of nuclear DNA^41,42^.

Coumaphos-treated patty causes considerable apoptosis in midgut tissue, which seems to be associated with accelerated bee mortality. Under laboratory conditions, imidacloprid exposure accelerates the removal or recycling (deletion) of apoptotic cells within the midgut, which reduces, or at worst, does not directly contribute to bee mortality. Additional research is needed to clarify the adaptive role of apoptosis in limiting tissue necrosis. Midgut tissues are not the intended targets of coumaphos and imidacloprid insecticides. Nevertheless, these two insecticides, and perhaps others as well, interact and alter midgut function, ultimately to the detriment of the bee. Interacting pesticide chemistries that cause unintended tissue and organ damage could lead to serious microbial infections and other comorbidities in honey bees^43^. Therefore, the underlying cytotoxic effects of coumaphos and imidacloprid on bee midgut epithelium must be considered when placing colonies in areas where these two insecticides are likely to enter hives simultaneously. Long-term exposure to realistic doses of imidacloprid induces bee mortality in laboratory tests and could, therefore, under field-conditions, reduce the size and productivity of stressed honey bee colonies^44^.

This study demonstrated synergy between insecticides and acaricides commonly used around honey bees, the effect of these chemicals on bee longevity, and also the harmful side-effects of these pesticides on bee midguts, which could lead to secondary microbial infections and further pollinator loss. Fortunately, the midgut of the honey bee can alleviate some of the cytotoxic effects of these compounds through selective apoptosis. More studies are needed to assess the effect and mode of action of sublethal concentrations of coumaphos and imidacloprid. We must also further examine synergistic effects of pesticides on pollinator tissues and organs at all levels of social structure (i.e. workers, drones, and queens).

## Materials and Methods

### Rearing conditions and treatments

**Figure 6:**
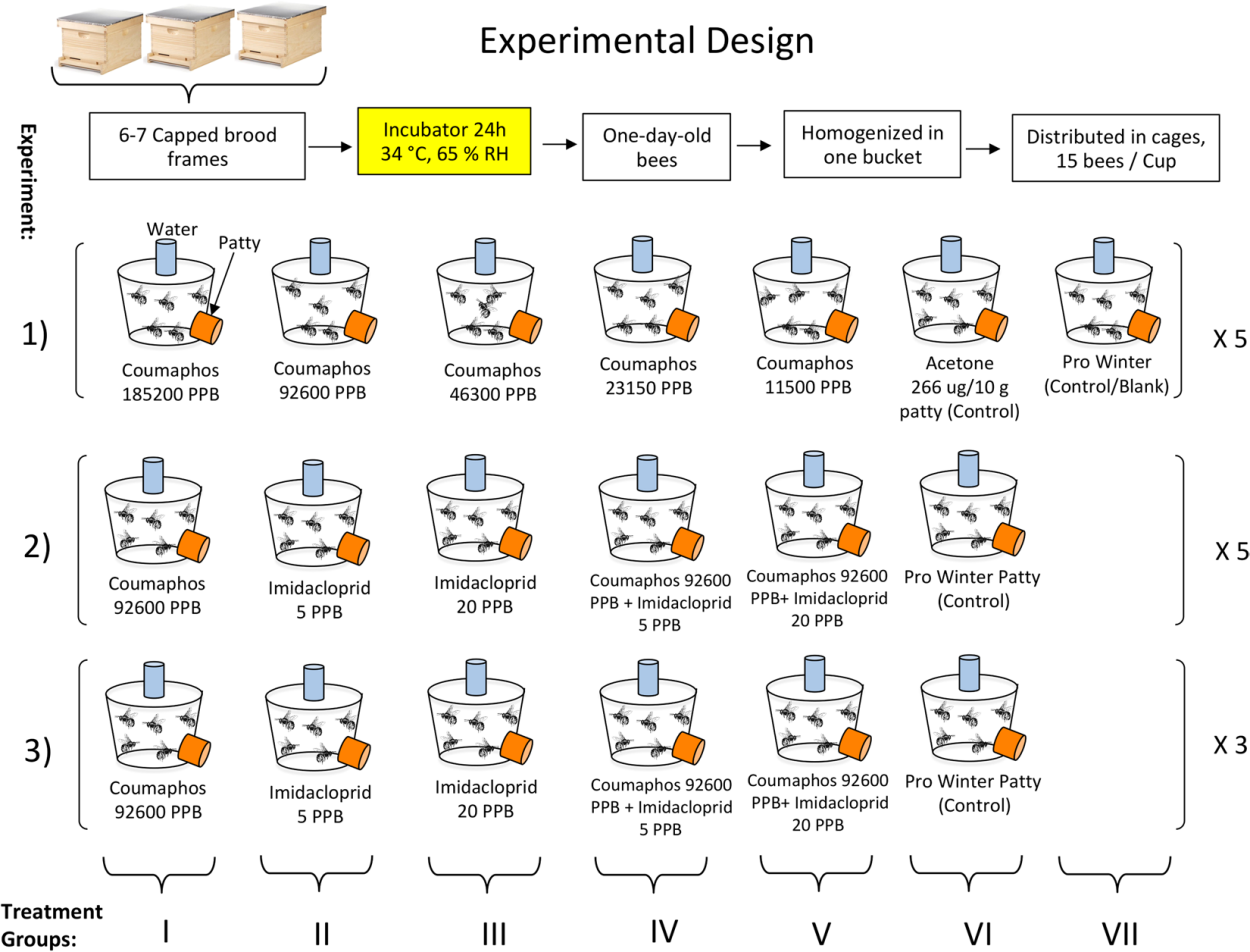
Experimental design (1–3) and the number of treatment groups in each experiment. Bee samples for the antioxidant gene study were collected from experiment 3. Additives to the diet were mixed to the Pro Winter patty (patty) as base sugar candy. Cages were also provided with distilled water. Experiments 1 and 2 contained five replicated cage groups; treatment 3 contained three replicated cage groups. Each cage per group housed 15 workers.

Experiments were conducted at the USDA-ARS Thad Cochran Southern Horticultural Research Laboratory in Poplarville, MS and at the Mississippi State University, Coastal Research and Extension Center, McNeill, MS. *Apis mellifera* workers used in these experiments emerged from brood combs removed from three honey bee colonies. Brood combs were incubated in a controlled environment at 34.5 °C and 65% RH in darkness. Newly emerged (one-day-old) bees were caged in plastic disposable cups (lower diameter 8 cm and height 13.5 cm) modified to serve as bioassay chambers or cages. These cages contained 80 aeration holes (diameter 3 mm) drilled into the side and one hole (diameter 12 mm) drilled on the top (the cup’s actual bottom) for the water supply and another port drilled on the side nearest the cup’s lid for removing dead bees and replenishing diet, Fig. 6. Bees in each group of cages were offered *ad libitum* water and the insecticides incorporated into Pro Winter sugar patties (patty) (Mann Lake Ltd.). Analytical standard-grade Fluka imidacloprid (Pestanal, CAS # 138261-41-3) and coumaphos (Pestanal, CAS # 56-72-4) were used as our insecticides. Treatment patties were prepared fresh and then stored in a refrigerator for the duration of the experiment. Three experiments were performed with treatments and sample sizes summarized in Fig. 6. Feeding experiments 1 and 2 were replicated five times, whereas, experiment 3 was replicated three times. The latter experiment served for both the antioxidant gene expression study and immunohistological tests on midgut tissue. Each cage (cup) contained 15 bees and 10 g of patty. When the patty was consumed, more was added. Dead bees in each cup were counted and removed daily.

In the first experiment, 5 concentrations of coumaphos (185,200; 92,600; 46,300; 23,150 and 11,500 ppb) were incorporated into the Pro Winter patty and five replicates for each treatment concentration group were conducted. Two treatment groups of caged bees, with five replicates in each, were used as a control treatment without the addition of coumaphos (VI, VII; Table 1 and Fig. 1). Control group (VI) was used to determine the toxicity of the coumaphos solvent (acetone). Therefore, for each 10 g patty, 266 µg of acetone was incorporated. The second ‘control/blank’ (VI) group consisted of the patty without insecticide or acetone. Coumaphos was dissolved using acetone and imidacloprid was dissolved in water at the same rates, for each 10 g patty, 266 µg acetone or water respectively. Dissolved substances were then gradually mixed in the needed patty volume.

In the second experiment, we tested bee mortality exposed to coumaphos and imidacloprid added to the Pro Winter sugar patty. Six treatment groups were established for this experiment detailed in Table 2 and Fig. 2. Coumaphos concentration used in this experiment was 92,600 ppb and concentrations for imidacloprid were 5 or 20 ppb. Mixtures of coumaphos and imidacloprid were administered to five replicates (cages) per treatment and one control group without pesticide. In experiments 1 and 2, we recorded food consumption across all treatment groups.

### Antioxidant gene expression associated with exposure to pesticides

Molecular and gene expression analyses were carried out at the University of Southern Mississippi, Department of Biological Sciences. This primary test was conducted to assess the effect of imidacloprid, coumaphos, and their combinations on a select set of honey bee antioxidant genes. For this study, 3 primary antioxidant enzymes, Catalase and two Superoxide Dimutases (SOD1 and SOD2) were chosen along with 7 secondary antioxidants that work to either recycle the primary antioxidants or act on the products of their reactions. These enzymes act directly on ROS (Reactive Oxygen Species) to alleviate oxidative stress and cellular damage. To conduct qPCR, worker bees were sampled from experiment 3. Three worker bees from each cage were sampled alive after feeding on the diets for 10 and 20 days. Sampled bees of each cage were preserved in 1.5 mL Eppendorf tubes and stored at – 80 °C. The target and reference genes are provided along with their NCBI accession numbers and primer sequences in Table 3. Worker bees sampled per cage were first manually homogenized via pestle in lysis buffer. The homogenized product was then quickly spun down and cellular debris removed using a sterilized needle. All proceeding steps for RNA extraction were carried out in accordance with the manufacturers provided protocol (GE Healthcare, illustar^TM^, RNAspin Mini Kit, Buckinghamshire, UK). RNA extractions were subsequently nanodropped (Thermo Scientific NanoDrop ND 1000 Spectrophotometers) for RNA quantity and quality and were set at 500 ng and stored at − 80 °C. Two-step reverse transcription quantitative PCR (RT-qPCR) was used to quantify the genetic expression of 10 antioxidant genes in bees. One microgram of RNA was used as a template for cDNA synthesis using Bio-Rad Kit (Iscript Kit, Bio-Rad). Each sample was run in triplicates using 15 ng of cDNA. RT-qPCR runs were conducted with LifeTechnologies PowerUP SYBR Green master mix using Bio-Rad C1000 Thermal Cycler. The target genes were normalized in all the RT-qPCR runs using a set of four housekeeping genes (Rp49, Rps5a, Tbp-f, RpL32) known to be stable and accurate in bee tissues^45^ (Table 3). Target genes were standardized against the housekeeping genes that adhere with acceptable stability standards^46^. It is worth noting that while most of our target genes are known to be linked to antioxidative activity in bees (Cat, Sod, Msr, TrX), we have added among our secondary antioxidant list, selenoprotein targets which are present in bees but poorly understood at the functional level.

**Table 3:** Identification, description, and NCBI accession numbers for both housekeeping and target genes.

Gene ID	Gene description	Accession ID	Primers F and R
**Housekeeping Genes**			
Rp49	Ribosomal Protein 49	AF441189	GTCACCAGAGTGATCGTTACA GGGCATCAAATATTGTCCCTTAAA
Rps5a	Ribosomal protein S5a	GB11132	GTACCTACCACGACGACATTA CACAATTCCAGCGACCAAATAA
Tbp-f	TATA box binding factor	XM_393492	GGAGGAGATACTCCAGCTATGTA CATCTGGTACCGTTGGTGTATAA
RpL32	Ribosomal protein L32	AF441189.1	GAGAAACTGGCGTAAACCTAAAG GTTGGCAACATATGACGAGTTT
**Target Genes**			
Primary Antioxidants			
Cat	Catalase	NP_001171540.1	TCCACTCATTCCTGTTGGTAAG GCCGGATCGAAGGCTATTT
Sod 1	Superoxide dismutase 1	NP_001171498.1	CGTTCCGTGTAGTCGAGAAAT GGTACTCTCCGGTTGTTCAAA
Sod 2	Superoxide dismutase 2	NP_001171519.1	TGCAGCAAGACGTATCCTATTT CATGGTGCTTTGAATGGTGAAG
Secondary Antioxidants			
MsrA	Methionine sulphoxide reductase A	NM_001178047.1	GGGCCGGTGATTGTTTATTTG CAACGACTTCTGTATGATCACCT
MsrB	Methionine sulphoxide reductase B	XM_006569172.1	GTATTAGATCAGGGACGAGTCAAG CATCCATCGTAGTTCTCTCCAA
Trxr1	Thioredoxin reductase	AY329357.1	CGTCCACCAACTCGTAGATTAG CTAGTACAACTTCTACATCCTCCAAA
SelK	Selenoprotein K-like	NM_001278332.1	CGTCCACCAACTCGTAGATTA CTAGTACAACTTCTACATCCTCCAAA
SelT	Selenoprotein T-like	XM_623426.5	ACAGCCACCAGCATCATT GGACCACACAGGAACATCATT
SelS	Small VCP/p97-interacting protein-like	XM_006559143.1	TGGGTGATGGTTCTAGAGGATA CACATTTCCTCAGCCTCGAATA
SelM	15 kDa selenoprotein M like protein	XM_006557387.2	CGATATCCACGTGCTGTTCT TCGGATCTAAACCTCTAACGTATTT

Sequences of forward (F) and reverse (R) primers used for each gene are provided in the fourth column.

### Immunohistological analyses

Worker bees from experiment 3 were sampled from four treatment groups (coumaphos 92,600 ppb, imidacloprid 20 ppb, coumaphos 92,600 ppb + imidacloprid 20 ppb, and untreated control) for immunohistological analyses. Three live workers from those mentioned groups were randomly sampled ten days after exposure to their respective pesticide compounds. Bee samples were anaesthetized by chilling for a few minutes. Abdomens were detached, midguts removed, and fixed in 10% formalin, dehydrated in ethanol, and embedded in paraffin wax. 5-µm sections of midgut tissue were mounted onto glass slides, deparaffinized, and then stained according to kit instructions: the ‘*In situ* cell death detection kit, AP’ (ISCDDK) (Roche, Mannheim, Germany; Cat. No. 11684809910) and the ‘ApopTag’ colorimetric apoptosis detection kit (Millipore Sigma, Cat. No. S7101). The Vector Red kit (Millipore Sigma, Cat. No. SK-5105) was used to obtain a red-colored precipitate in sections submitted to ISCDDK assay. Sections were counterstained with hematoxylin. TUNEL-positive cells appeared red, which are indicated by a reaction product localized in the nucleus, which shows a dying cell (cell death). TUNEL-negative nuclei of healthy cells appeared blue. Histological sections submitted to ApopTag *in situ* apoptosis detection kit were incubated with diaminobenzidine tetrahydrochloride (DAB). The sections were counterstained with hematoxylin. TUNEL-positive cells appeared brown, which are indicated by a reaction product localized to the nucleus and indicative of cell death. TUNEL-negative nuclei of healthy cells appear blue.

Cell death was detected using TUNEL and terminal deoxynucleotidyl transferase (TdT)-mediated dUTP for DNA labelling, and anti-fluorescein alkaline phosphatase conjugated antibody (ISCDDK), fast red was used for visualization, and counterstaining with haematoxylin. In both cell death assays, a control label was established by substituting the deoxynucleotidyl transferase (TdT) enzyme with phosphate buffered saline (PBS) during the TUNEL reaction. Sections were mounted in Faramount aqueous mounting medium (Dako). All slides were digitally photo-documented through a bright field light microscope.

### Data analyses

Survivorship curves were generated for each cage among a given treatment using the LifeReg procedure in SAS version 9.4^47^. For both cage experiments, a Weibull distribution described the data most appropriately. With respect to the 30, 50, and 70^th^ percentile, treatments were compared using ANOVA from the Glimmix procedure in SAS version 9.4^47^ and figures generated using SigmaPlot (https://systatsoftware.com). Mean separation for the consumption data was generated using Tukey test.

The gene expression study was conducted using CFX Maestro Software for Bio-Rad Real-time PCR Systems. All target genes were standardized using four housekeeping genes that exhibited acceptable stability values; Coefficient Variance stability <0.5 and mean value <1^45^. This was always the case except for one study set in which (RPS5a) was excluded from the analysis as it failed to meet those standards. Data were analyzed using normalization expression (ΔΔCq) and target genes with Cq values that did not have a P value smaller that 0.05 (Normalization threshold) were not considered up or down-regulated. Chart error bars across the data are the standard error SE. Volcano plots, Bar charts, and Clustergram were all generated using CFX Maestro Software while conducting the gene expression study.

Semi-quantitative analysis of cell death TUNEL-labeled tissue slides were used for quantification of cell type and impending cell death using ISCDDK and ApopTag kits. For each experimental group of bees, approximately 300 total cells from each individual bee were counted in random fields on different slides. The results were expressed as the proportion of cells with positive staining. To confirm reproducibility, 25% of the slides were chosen randomly and scored twice. The proportion of cells with positive staining was converted into scores, based on established protocols^48^.

## Electronic supplementary material

Figure S1
