# Supplementary material for: Effects of coumaphos and imidacloprid on honey bee (Hymenoptera: Apidae) lifespan and antioxidant gene regulations in laboratory experiments
Source: Sci Rep. 2018 Oct 9;8:15003. doi: 10.1038/s41598-018-33348-4 (PMC6177410; doi:10.1038/s41598-018-33348-4)

Effects of coumaphos and imidacloprid on honey bee (Hymenoptera: Apidae) lifespan and antioxidant gene regulations in laboratory experiments

Ales Gregorc[1,2]*, Mohamed Alburaki[3], Nicholas Rinderer[3], Blair Sampson[4], Patricia R. Knight[1], Shahid Karim[3], John Adamczyk[4]

[1]Mississippi State University, Center for Costal Horticulture Research, Poplarville, MS, USA

[2]Agricultural Institute of Slovenia, Ljubljana, Slovenia and University of Maribor, Faculty of Agriculture and Life Sciences, Maribor, Slovenia

[3] The University of Southern Mississippi, Department of Biological Sciences, Hattiesburg, MS, USA

[4]USDA, ARS, Thad Cochran Southern Horticultural Research Laboratory, Poplarville, MS, USA

Authors' contacts:

Ales Gregorc: ales.gregorc@kis.si
Mohamed Alburaki : mohamed.alburaki@usm.edu
Nicholas Rinderer: nicholas.Rinderer@usm.edu
Blair Sampson: blair.sampson@ars.usda.gov
Patricia R. Knight: prk3@msstate.edu
Shahid Karim: shahid.karim@usm.edu
John Adamczyk: john.adamczyk@ars.usda.gov

*Corresponding author. E-mail: ales.gregorc@kis.si (A. Gregorc).

Figure S1. Illustration of the primary and secondary antioxidants activity in event of oxidative stress such as exposure to pesticides. Details are given on the role of seleno-like proteins as regulators in reducing oxidative stress in the endoplasmic reticulum ER and maintaining protein quality.

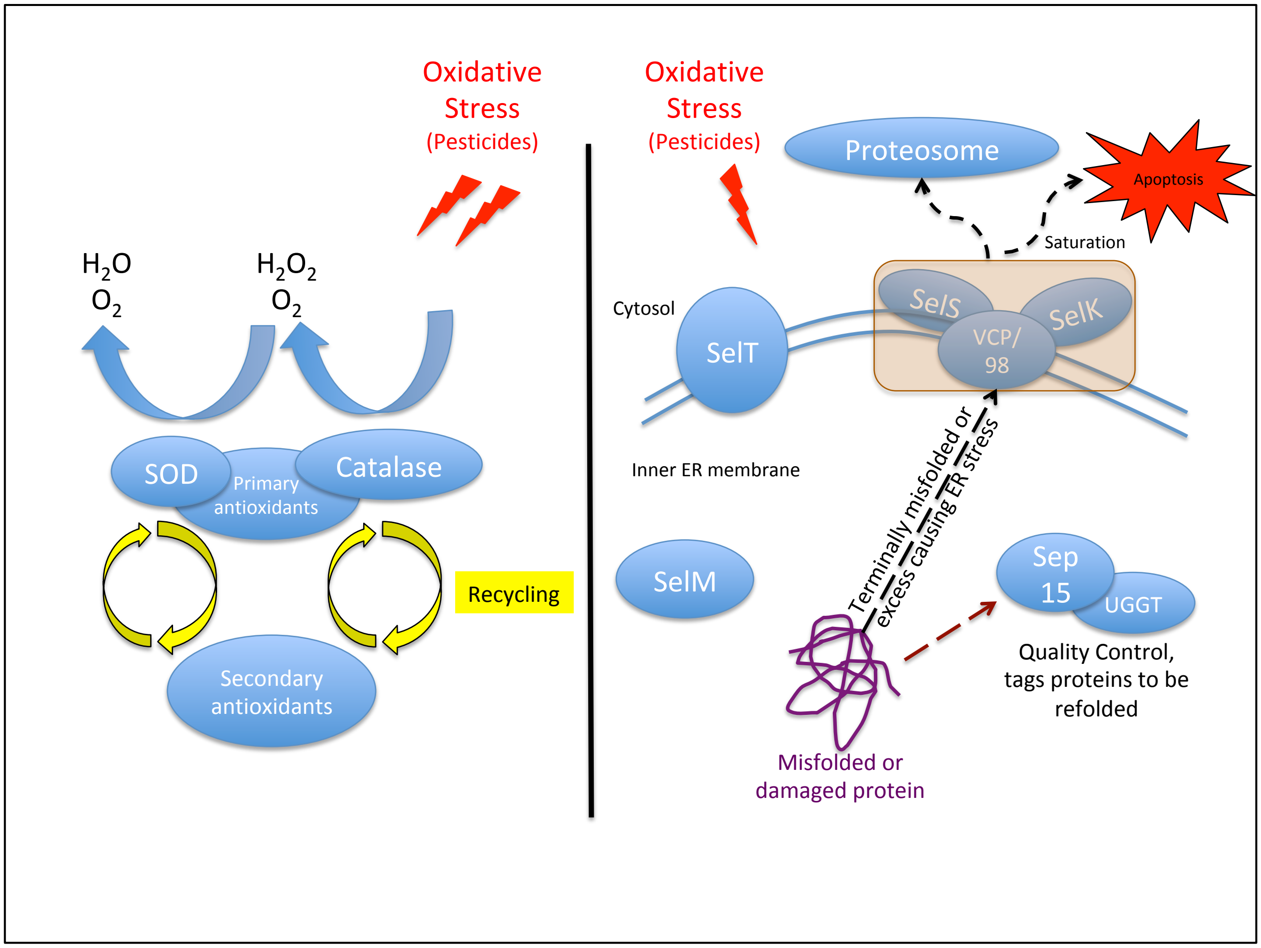

Supplement: Supplementary file 1 — Figure S1 [file 41598_2018_33348_MOESM1_ESM.pdf]
